# Protective Role of Perivascular Adipose Tissue in Endothelial Dysfunction and Insulin-Induced Vasodilatation of Hypercholesterolemic LDL Receptor-Deficient Mice

**DOI:** 10.3389/fphys.2018.00229

**Published:** 2018-03-19

**Authors:** Natali Baltieri, Daniele M. Guizoni, Jamaira A. Victorio, Ana P. Davel

**Affiliations:** Department of Structural and Functional Biology, Institute of Biology, University of Campinas, Campinas, Brazil

**Keywords:** perivascular adipose tissue, endothelium, LDL receptor deficiency, hypercholesterolemia, nitric oxide, insulin, adiponectin

## Abstract

**Background:** Endothelial dysfunction plays a pivotal role in the initiation of atherosclerosis. Vascular insulin resistance might contribute to a reduction in endothelial nitric oxide (NO) production, leading to impaired endothelium-dependent relaxation in cardiometabolic diseases. Because perivascular adipose tissue (PVAT) controls endothelial function and NO bioavailability, we hypothesized a role for this fat deposit in the vascular complications associated with the initial stages of atherosclerosis. Therefore, we investigated the potential involvement of PVAT in the early endothelial dysfunction in hypercholesterolemic LDL receptor knockout mice (LDLr-KO).

**Methods:** Thoracic aortas with and without PVAT were isolated from 4-month-old C57BL/6J (WT) and LDLr-KO mice. The contribution of PVAT to relaxation responses to acetylcholine, insulin, and sodium nitroprusside was investigated. Western blotting was used to examine endothelial NO synthase (eNOS) and adiponectin expression, as well the insulin signaling pathway in aortic PVAT.

**Results:** PVAT-free aortas of LDLr-KO mice exhibited impaired acetylcholine- and insulin-induced relaxation compared with those of WT mice. Both vasodilatory responses were restored by the presence of PVAT in LDLr-KO mice, associated with enhanced acetylcholine-induced NO levels. PVAT did not change vasodilatory responses to acetylcholine and insulin in WT mice, while vascular relaxation evoked by the NO donor sodium nitroprusside was not modified by either genotype or PVAT. The expression of insulin receptor substrate-1 (IRS-1), phosphatidylinositol 3-kinase (PI3K), AKT, ERK1/2, phosphorylation of AKT (Ser473) and ERK1/2 (Thr202/Tyr204), and adiponectin was similar in the PVAT of WT and LDLr-KO mice, suggesting no changes in PVAT insulin signaling. However, eNOS expression was enhanced in the PVAT of LDLr-KO mice, while eNOS expression was less abundant in PVAT-free aortas.

**Conclusion:** These results suggest that elevated eNOS-derived NO production in aortic PVAT might be a compensatory mechanism for the endothelial dysfunction and impaired vasodilator action of insulin in hypercholesterolemic LDLr-deficient mice. This protective effect may limit the progression of atherosclerosis in genetic hypercholesterolemia in the absence of an atherogenic diet.

## Introduction

Hypercholesterolemia is the main risk factor for the development of atherosclerosis by enhancing low-density lipoprotein (LDL) retention within the vessel wall (Stapleton et al., 2010). Atherosclerosis is a progressive disease in which inflammation, fat deposits, and cells and extracellular matrix accumulation in the artery result in the occlusion of the vessel lumen, underlying a major cause of clinical cardiovascular events worldwide (World Health Organization, 2014; Mozaffarian et al., 2016). One of the initial steps of the atherosclerotic process is the development of endothelial dysfunction, which precedes atherosclerosis development in humans (Guzik et al., 2000; Heitzer et al., 2001).

LDL receptor knockout mice (LDLr-KO) is a model of human familial hypercholesterolemia (FH). Although young adult LDLr-KO mice fed with standard low-fat diet only develop small spontaneous atherosclerotic lesions in the aortic root (Dorighello et al., 2016, 2017), these animals present impaired endothelium-dependent relaxation in the aorta (Rabelo et al., 2003; Langbein et al., 2015; Guizoni et al., 2016). This finding is consistent with reduced endothelium-dependent vasodilation in human hypercholesterolemia (Creager et al., 1990, 1992). In this model of genetic hypercholesterolemia, endothelial dysfunction is associated with the reduced gene and protein expression of endothelial nitric oxide synthase (eNOS) (Guizoni et al., 2016; Langbein et al., 2016), and reduced dimerization of this enzyme, resulting in impaired endothelial nitric oxide (NO) release (Guizoni et al., 2016).

Nitric oxide (NO) is an endothelium-derived vasodilator factor with antiatherogenic properties, including the inhibition of platelets, and monocyte adhesion to the endothelium, platelet aggregation, smooth muscle proliferation, and oxidation of LDL (Vanhoutte, 2003). Consistently, the administration of L-arginine, as eNOS substrate and NO precursor, improves the endothelial function in hypercholesterolemic patients (Creager et al., 1992; Siasos et al., 2007), whereas the lack of NO accelerates the progression of atherosclerosis (Kauser et al., 2000). Genetic hypercholesterolemia is also associated with the impaired secretion of insulin (Souza et al., 2013), a hormone stimulating NO production (Potenza et al., 2009). In addition to endothelium-derived NO, NO release from perivascular adipose tissue (PVAT) has recently been demonstrated as an important adipocyte-derived relaxation factor (ADRF) in the aorta (Victorio et al., 2016; Xia et al., 2016), but whether or not changes in PVAT eNOS function are involved in hypercholesterolemia-induced vascular dysfunction remains unknown.

Perivascular adipose tissue (PVAT) surrounds most blood vessels and releases numerous factors and adipokines with paracrine effects on both vascular structure and function (Akoumianakis et al., 2017). Higher levels of proinflammatory adipocytokines and lower levels of adiponectin were found within the PVAT after artery injury (Takaoka et al., 2010), and an inflamed PVAT results in neointima formation (Moe et al., 2013). In addition, increased amounts of angiogenic factors release by PVAT may have a pathological relevance for atherosclerosis development (Chang et al., 2013). However, the thermogenic properties of PVAT have been demonstrated as anti-atherogenic (Chang et al., 2012; Brown et al., 2014). Nevertheless, the role of PVAT in the development and progression of this vascular disease is still unclear. Because PVAT regulates endothelial function and NO bioavailability, we hypothesized a role for this fat deposit in the vascular complications associated with the initial stages of atherosclerosis. Therefore, the present study was designed to evaluate the potential influence of PVAT in the early endothelial dysfunction of hypercholesterolemic LDLr-KO mice.

## Materials and methods

### Animals

LDL receptor knockout (LDLr-KO) mice and respective C57BL6/J wild-type (WT) mice were purchased from the Jackson Laboratory, and the strains are maintained by breeding at the Multidisciplinary Center for Biological Research (CEMIB-UNICAMP, Campinas, SP, Brazil) with genotypic control. The mice were housed at 22 ± 1°C on a 12:12 h light:dark cycle with free access to a standard rodent chow diet (Nuvital CR1, Colombo, Paraná, Brazil) and water. At 4 months old, the mice were weighted and anesthetized with urethane (5 g/kg body weight, i.p.) to collect blood samples and isolate thoracic aorta.

All experimental protocols were approved (protocol # 3639-1) by the Ethics Committee on Animal Use of the University of Campinas (CEUA-UNICAMP, Campinas-SP, Brazil) and carried out in accordance with the ethical principles for animal experimentation adopted by the Brazilian Society of Laboratory Animal Science (SBCAL/COBEA).

### Plasma biochemical analysis

Blood samples were obtained from the tail vein for blood glucose measurement (Accu-Chek Advantage, Roche Diagnostics, Sao Paulo, Brazil). Subsequently, the mice were anesthetized (5 g/kg of urethane, i.p.), and blood samples were collected by cardiac puncture and centrifuged (8,000 g for 15 min at 4°C); the serum supernatants were subsequently collected for biochemical analysis. Total cholesterol and triglyceride levels were measured using standard commercial kits (Chod-Pap, Roche Diagnostic GmbH, Mannheim, Germany).

### Vascular reactivity in the presence or absence of PVAT

The thoracic aorta was isolated and cut into cylindrical segments (~2 mm in length) with or without surrounding PVAT. The segments were mounted in a tissue chamber bath (Panlab Harvard Apparatus, Cornellà-Barcelona, Spain) containing Krebs–Henseleit solution (in mM: 118 NaCl, 4.7 KCl, 25 NaHCO3, 2.5 CaCl2-2H2O, 1.2 KH2PO4, 1.2 MgSO4-7H2O,11 glucose and 0.01 EDTA; pH = 7.4, 37°C) with a resting tension of 0.5 g stabilized for 1 h, as previously demonstrated (Davel et al., 2012; Guizoni et al., 2016). Subsequently, aortic rings were exposed to 125 mM of KCl to test vascular integrity and assess maximal contraction, and no differences were found among groups (data not shown). Following washing, the aortic rings were contracted with a submaximal concentration of the thromboxane A2 receptor agonist (U-46619, 70% of maximal contraction to 125 mM of KCl) and relaxation curves to acetylcholine (0.1 nmol/L to 10 μmol/L, Sigma-Aldrich, Saint Louis, MO, USA), insulin (0.1 to 10 nmol/L, Humulin® R - rDNA origin, Lilly USA, Indianapolis, USA), or the NO-donor sodium nitroprusside (1 pmol/L to 0.1 μmol/L Sigma-Aldrich, Saint Louis, MO, USA) were performed.

### PVAT homogenization and western blotting

Thoracic aorta and respective PVAT were separately pulverized in N2 liquid and homogenized in cold RIPA lysis buffer (Merck Millipore, Billerica, MA, USA) containing PMSF (1 mM), Na3VO4 (1 mM) and 2 μL/mL of protease inhibitor cocktail (PIC, Sigma-Aldrich) to obtain total protein extract (Victorio et al., 2016). The proteins were quantified using the BCA Protein Assay Kit (Thermo Fisher Scientific Inc.) to determine the protein concentration values of the samples (PVAT: *WT* = 4.49 ± 2.94 and LDLr-KO = 5.74 ± 1.98 mg/ml; aorta: *WT* = 6.13 ± 3.09 and LDLr-KO = 7.46 ± 1.98 mg/ml). Then, 40 μg of PVAT or aorta extracts and the 1 μL of plasma were electrophoretically separated on 7.5 or 12% acrylamide SDS–PAGE and then transferred to PVDF membranes using a Mini Trans-Blot Cell system. Non-specific binding sites were blocked with 5% non-fat milk solution, and then the membranes were incubated for 12 h at 4°C with the following primary antibodies: anti-insulin receptor substrate-1 (IRS-1), anti-p85 subunit of phosphatidylinositol 3 kinase (PI3K), anti-Akt1/2/3, anti-phospho (Ser473)-Akt1/2/3 (1:1,000; Santa Cruz Biotechnology), anti-eNOS (1:1,000; BD Transduction, Franklin Lakes, NJ, USA), anti-ERK1/2, anti-phospho (Thr202/Tyr204)-ERK1/2 (1:1,000; Cell Signaling, Danvers, MA, USA), and anti-adiponectin (1.5 μg/mL, Novus Biologicals, Littleton, CO, USA). Additionally, α-tubulin (1:1,000; Santa Cruz Biotechnology) was used as an internal control protein for PVAT, α-actin (1:1,000; Abcam, Cambridge, MA, USA) was used as an internal control protein for aorta, and Ponceau S staining for adiponectin expression in plasma and PVAT. After washing (10 mM of Tris, 100 mM of NaCl and 0.1% Tween-20), the membranes were incubated for 90 min with the specific secondary antibody conjugated to peroxidase. Immunocomplexes were detected using a luminol peroxidase chemiluminescence kit (ECL Plus, Amersham) and visualized using photographic film (Hyperfilm ECL, Amersham) in a dark room. The intensity of the immunoblots was quantified using ImageJ 1.46p software (National Institutes of Health, Bethesda, MD, USA).

### PVAT NO release

NO in PVAT was detected with 4,5-diaminofluorescein diacetate (DAF-2 DA) (Xia et al., 2016). Aortic PVAT cryostat sections (20 μm) were loaded with DAF-2 DA (8 μM) in the presence of acetylcholine (10 μM) at 37°C for 30 min. Then, fluorescence imaging was performed with a microscope (Eclipse Ti-S, Nikon, Tokyo, Japan) equipped with a fluorescence filter. DAF-2 DA was excited by an interference filter at 465–495 nm, and fluorescence emitted between 515 and 555 nm was collected (Zhou and He, 2011). The images captured under a 10X objective were analyzed using ImageJ 1.46p software (National Institutes of Health), the mean optical density of the fluorescence was measured, and the results were normalized by the PVAT area (μm^2^). Fluorescence intensity was quantified by the subtraction of acetylcholine-treated samples from basal conditions.

### Statistical analysis

Data were expressed as the means ± SEM. Two-way ANOVA was used to analyze the vasorelaxation response curves. When ANOVA showed a significant effect, Bonferroni's *post-hoc* test was used to compare individual means. Unpaired Student's *t*-test was used for two-group comparisons. For each concentration-response curve, *R*_max_ and the negative logarithm of the concentration of the agonist that produced half of *R*_max_ (−LogEC_50_) were calculated using non-linear regression analysis. GraphPad Prim Software 5.0 (San Diego, CA, EUA) was used for statistical analysis and −LogEC_50_ and *R*_max_ calculation. *P* < 0.05 values were considered significant.

## Results

As expected, LDLr-KO mice exhibited dyslipidemia characterized by elevated plasma levels of total cholesterol and triglycerides (Bonfleur et al., 2011), with no changes in blood glucose or body weight (Table 1).

**Table 1:** Body weight and total serum cholesterol, triglycerides, and glycemia in wild-type (WT) and LDLr knockout mice (LDLr-KO).

	**WT**	**LDLr-KO**
Body weight (g)	28 ± 0.6	27 ± 0.6
Blood glucose (mg/dL)	135 ± 14	143 ± 23
Total cholesterol (mg/dL)	84.6 ± 4.6	262.3 ± 14.3^*^
Triglycerides (mg/dL)	54.3 ± 4.1	145.4 ± 11.5^*^

Data are expressed as mean ± SEM (N = 10–14). Unpaired Student's t-test:

* *P < 0.05 vs. WT*.

**Figure 1:**
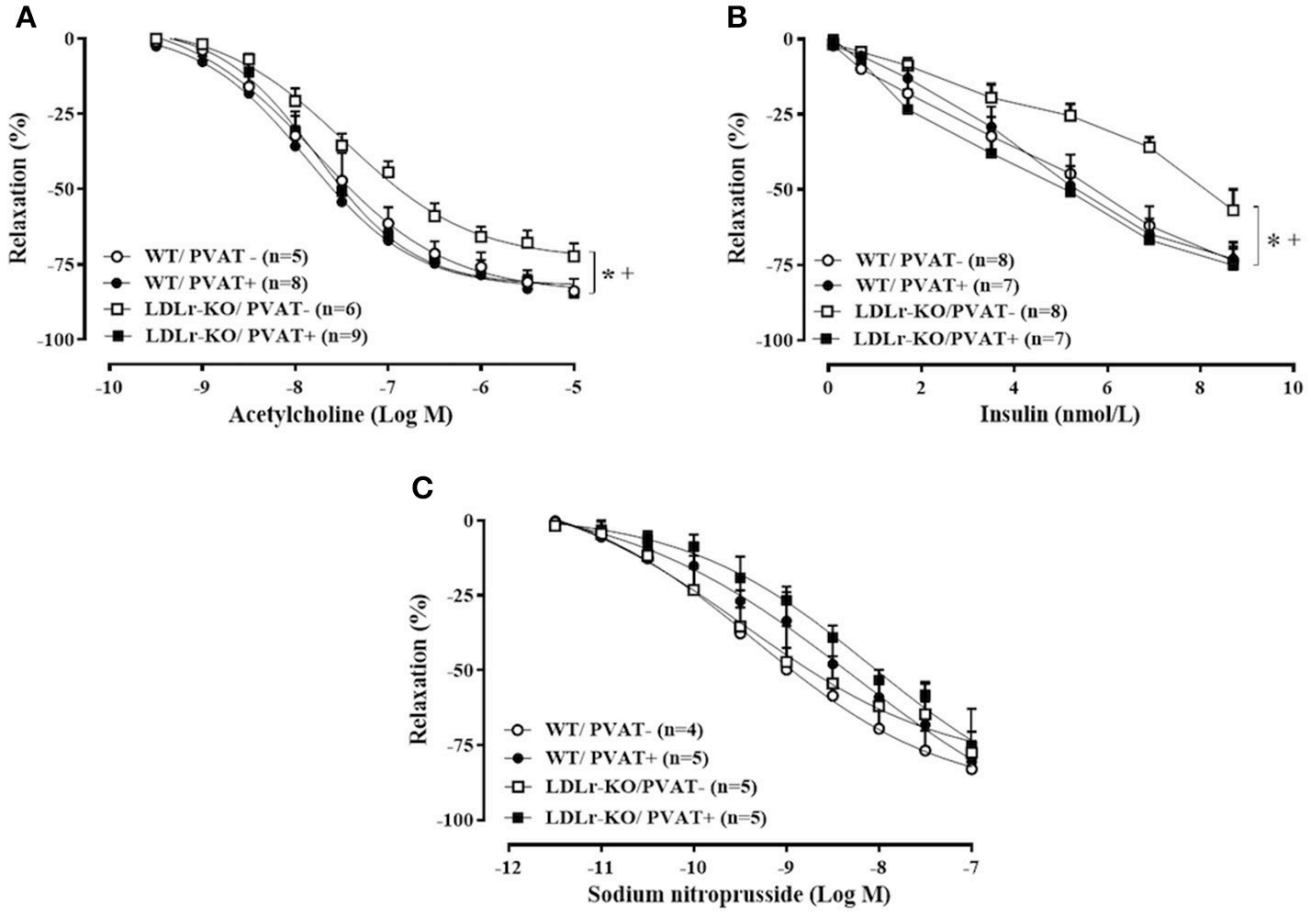
Perivascular adipose tissue (PVAT) avoids endothelial dysfunction in LDLr knockout mice. Relaxation curves to acetylcholine **(A)**, insulin **(B)**, and sodium nitroprusside **(C)** in aortic rings with (+) and without (–) PVAT from wild-type (WT; circles) and LDLr knockout mice (LDLr-KO; squares). Two-way ANOVA, *P* < 0.05: * vs. WT/PVAT–; ^+^ vs. LDLr-KO/PVAT+.

The vascular reactivity study revealed an impaired relaxation to acetylcholine and insulin in aorta of LDLr-KO mice in the absence of PVAT (Figures 1A,B), with a reduced *R*_max_ to both agonists (Table 2). These data suggest an impaired endothelial vasodilatory response and vascular insulin resistance. However, the presence of PVAT prevented the impaired vasodilatory response to both acetylcholine and insulin in LDLr-KO associated with increased *R*_max_ while not affecting vasorelaxation in WT (Figures 1A,B; Table 2). Changes in acetylcholine- and insulin-induced relaxation were not the result of a smooth muscle defect, as the relaxation response to the NO donor sodium nitroprusside was similar between WT and LDLr-KO mice, with or without PVAT (Figure 1C, Table 2).

**Table 2:** Maximal relaxation response (R_max_) and potency for acetylcholine, insulin, and sodium nitroprusside in aortas with (+) or without (–) perivascular adipose tissue (PVAT) from wild-type (WT) and LDLr knockout mice (LDLr-KO).

	**WT**		**LDLr-KO**	
	**PVAT−**	**PVAT+**	**PVAT−**	**PVAT+**
**ACETYLCHOLINE**				
R_max_ (%)	84 ± 4.1	87 ± 3.9	70 ± 4.7^*^	85 ± 1.3^+^
−LogEC_50_	7.67 ± 0.16	7.81 ± 0.16	7.37 ± 0.14	7.74 ± 0.11
**INSULIN**				
R_max_ (%)	76 ± 3.9	73 ± 4.0	57 ± 6.9^*^	76 ± 4.1^+^
−LogEC_50_	8.39 ± 0.06	8.39 ± 0.05	8.30 ± 0.03	8.48 ± 0.09
**SODIUM NITROPRUSSIDE**				
R_max_ (%)	83 ± 4.9	80 ± 6.9	78 ± 14.7	75 ± 4.8
−LogEC_50_	9.33 ± 0.49	9.04 ± 0.17	9.15 ± 0.28	8.51 ± 0.08

Data are expressed as mean ± SEM (N = 4–9). 2-way ANOVA followed by Bonferroni's post-hoc test, P < 0.05.

* vs. WT/PVAT-;

+ *vs. LDLr-KO/PVAT−*.

**Figure 2:**
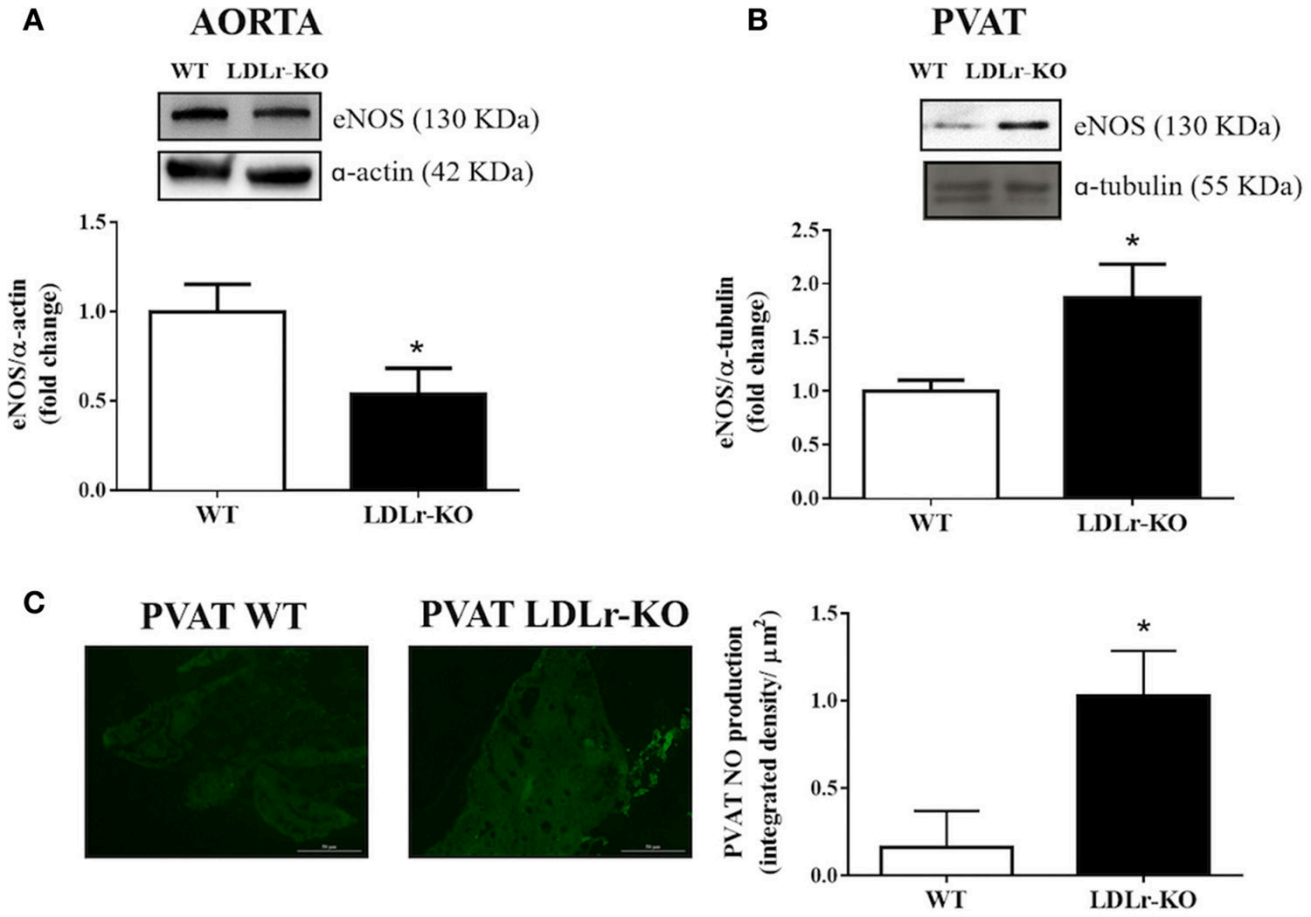
eNOS expression is reduced in aorta while is increased in perivascular adipose tissue (PVAT) of LDLr knockout mice. Aortic **(A)** and PVAT **(B)** eNOS expression in wild-type (WT) and LDLr knockout (LDLr-KO) mice. Representative blots are shown at the top of the graphs. **(C)** Representative images and the quantification of PVAT NO production determined by DAF-2 DA fluorescence intensity in response to acetylcholine in WT and LDLr-KO mice. Student's *t*-test, *P < 0.05 vs. WT.

Considering that NO is the main endothelium-derived vasodilator factor mediating acetylcholine- and insulin-induced relaxation in the murine aorta (Wu et al., 1994), we investigated vascular and PVAT eNOS expression. Interestingly, while eNOS expression was reduced in aortic tissue from LDLr-KO mice (Figure 2A), aortic PVAT showed more abundant eNOS expression (Figure 2B), associated with enhanced PVAT NO levels (Figure 2C). These data suggest increased eNOS-derived NO production as a mechanism involved in the protective effect of aortic PVAT in dyslipidemic LDLr-KO mice.

**Figure 3:**
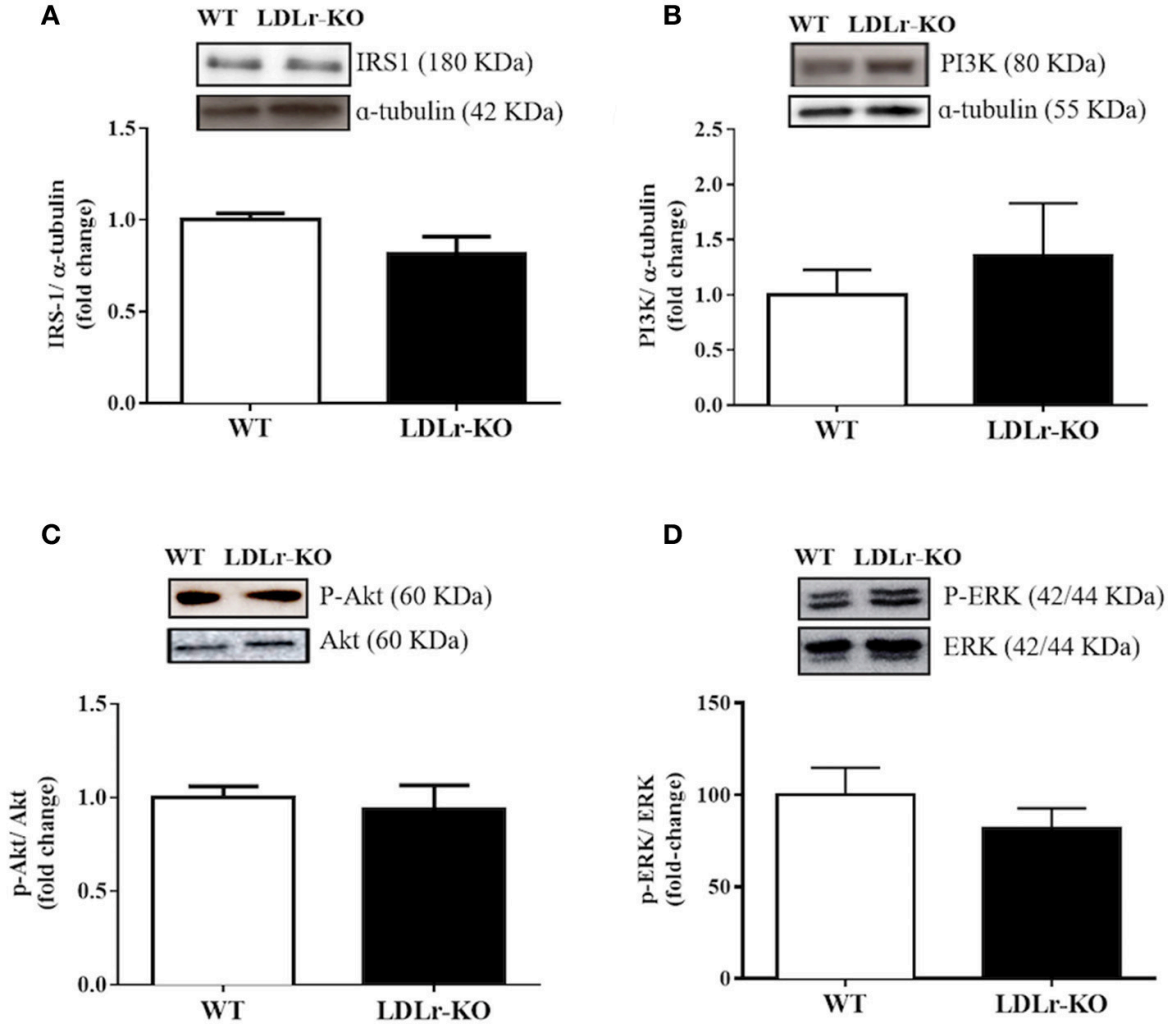
Vascular insulin signaling has not changed in perivascular adipose tissue (PVAT) of LDLr knockout mice. Protein expression of IRS-1 **(A)**, p85 subunit of PI3K **(B)**, p-Akt/Akt ratio **(C)**, and p-ERK/ERK ratio **(D)** in PVAT from wild-type (WT) and LDLr knockout (LDLr-KO) mice. Representative blots are shown at the top of the graphs.

To investigate insulin signaling in PVAT, we evaluated the protein expression of IRS-1, PI3K, and total and phosphorylated Akt and ERK. However, as shown in Figure 3, no differences between groups were observed.

**Figure 4:**
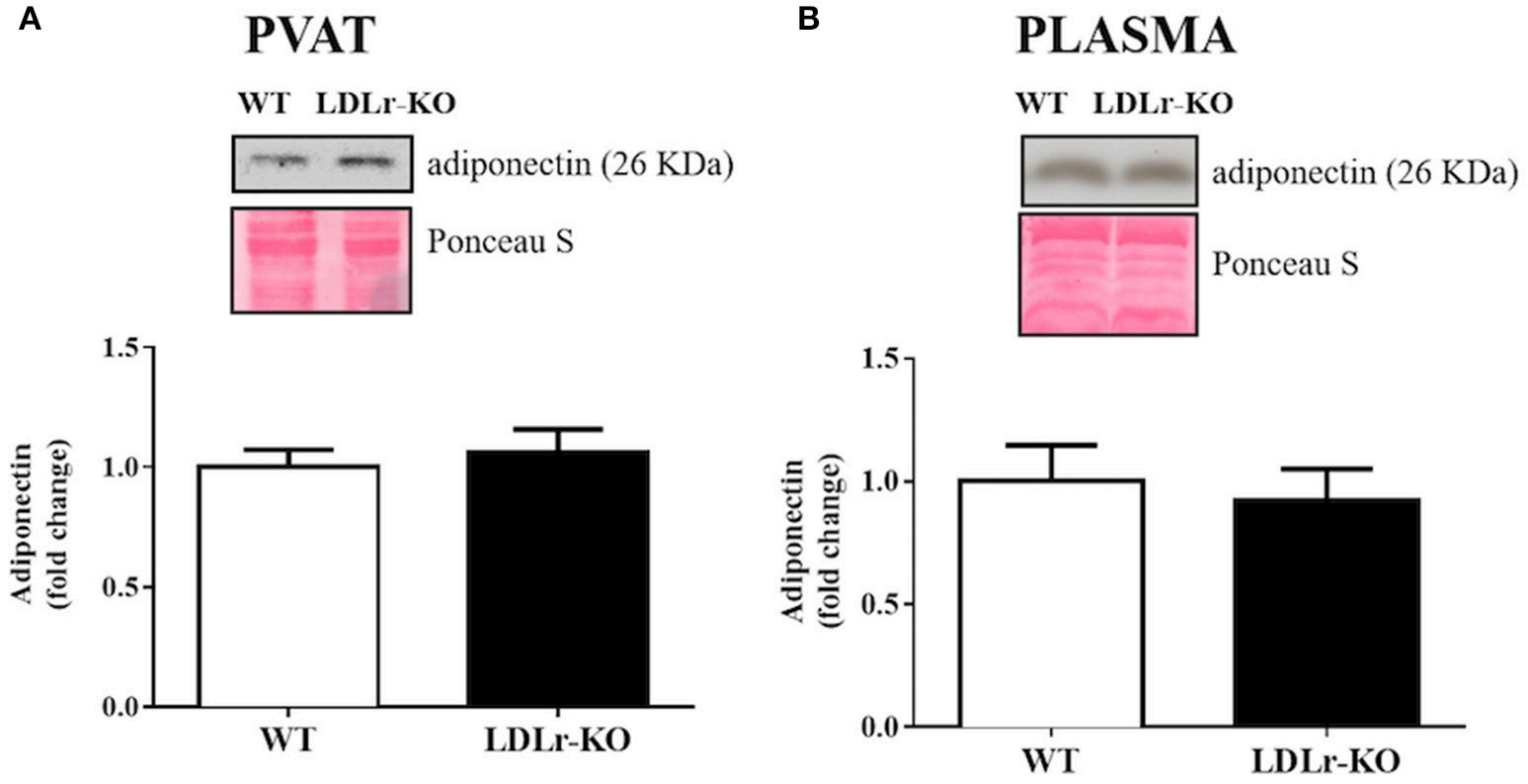
Circulating and perivascular adiponectin expression. Protein expression of adiponectin in aortic perivascular adipose tissue (PVAT) **(A)** and in plasma **(B)** from wild-type (WT), and LDLr knockout (LDLr-KO) mice. Representative blots are shown at the top of the graphs.

Finally, because adiponectin is an important adipokine that stimulates NO production and facilitates endothelium-dependent relaxation (Margaritis et al., 2013), we examined PVAT and circulating adiponectin expression (Figures 4A,B). No significant changes were found between WT and LDLr-KO mice.

## Discussion

The results from the present study showed that in LDLr-KO mice, the presence of PVAT protected against impaired endothelium-dependent relaxation to acetylcholine and insulin, in association with enhanced eNOS protein expression and NO levels in thoracic aortic PVAT. Since this genetic model of FH exhibited only small aortic-root lesions in mice maintained on a standard low-fat diet (Dorighello et al., 2016), these data reveal a protective role for PVAT in an early phase of vascular injury induced by hypercholesterolemia.

We (Guizoni et al., 2016) and others (Rabelo et al., 2003; Langbein et al., 2015) have demonstrated a slight but significant reduction in acetylcholine-induced relaxation in LDLr-KO mice, as evidenced by a reduced maximal response to this agonist (Hofmann et al., 2017). Similarly, we found a reduced maximal relaxation to acetylcholine in aorta without PVAT from LDLr-KO fed a standard diet, indicating endothelial dysfunction in this genetic model of FH. This endothelial dysfunction became more evident in the presence of high fat/high cholesterol diets or the upregulation of lectin-like oxidized LDLr-1 (LOX-1) (Hofmann et al., 2017). However, Western-type diets also induced a range of secondary factors, such as inflammation, insulin resistance, and obesity, which synergistically interact to increase atherosclerosis. A high-fat diet per se upregulates proinflammatory gene expression in PVAT depots (Chatterjee et al., 2009). Inflamed fat surrounding the vessels resulted in enhanced atherosclerotic lesions and exacerbated endothelial dysfunction (Öhman et al., 2011). Therefore, PVAT may have pathological relevance in advanced atherosclerosis, contributing to plaque complications (Chang et al., 2013). In the present study, we investigated the role of PVAT in endothelium-dependent relaxation in the early stages of vascular injury in LDLr-KO mice fed a standard diet. Interestingly, the presence of PVAT improved relaxation responses to acetylcholine and insulin in the aortas of LDLr-KO mice, suggesting a protective role for this tissue in the present genetic model of FH by improving endothelial function. In healthy mouse aorta, the presence of PVAT did not affect the endothelium-dependent relaxation induced by acetylcholine, as previously demonstrated (Ketonen et al., 2010; Li et al., 2011).

Gil-Ortega et al. (2010) showed adaptive NO overproduction in PVAT during the initial steps of high-fat diet-induced obesity in mice. PVAT-derived NO may contribute to the anticontractile effect of PVAT independently of the endothelium (Aghamohammadzadeh et al., 2016). Since eNOS is expressed in aortic endothelium and PVAT (Victorio et al., 2016; Xia et al., 2016), we investigated whether the protective role of PVAT in endothelium-dependent relaxation responses in LDLr-KO mice was associated with changes in eNOS expression. The results demonstrated reduced eNOS expression in the vascular wall of LDLr-KO mice, as previously demonstrated, associated with reduced NO production (Guizoni et al., 2016). Lower eNOS and nNOS activation is also related to endothelial dysfunction in apolipoprotein E-deficient mice (Capettini et al., 2011). In accordance with these data, lower NO production is observed in endothelial cells exposed to the serum of hypercholesterolemic patients (Feron et al., 1999). However, in contrast to lower endothelial eNOS expression in the vessel wall, increased eNOS expression and NO levels were observed in the aortic PVAT of LDLr-KO mice. Therefore, higher eNOS-derived NO production in surrounding fat may be the mechanism enhancing endothelium-dependent relaxation in the aortas of LDLr-KO mice.

In addition to reduced relaxation to acetylcholine, the aortas from LDLr-KO mice without PVAT exhibited impaired relaxation to insulin. Insulin is an important stimulus in endothelial cells for eNOS-derived NO production (Potenza et al., 2009). Therefore, lower eNOS expression in the vascular wall should be a mechanism involved in the impaired relaxation to this hormone in hypercholesterolemic mice. Since relaxation response to sodium nitroprusside was not different among groups, changes in smooth muscle responsiveness may not be involved in the reduced vasodilatory responses observed in LDLr-KO mice. High plasma levels of cholesterol in LDLr-KO mice impairs beta-cell pancreatic function reducing insulin secretion, even in the absence of metabolic factors induced by Western-type diets, suggesting that genetic hypercholesterolemia increases the risk of diabetes development (Bonfleur et al., 2011; Souza et al., 2013). This finding is consistent with the slight increase in fasted plasma glucose previously observed in LDLr-KO mice (Bonfleur et al., 2011; Souza et al., 2013), although we did not find significant differences in non-fasted animals. In the present study, the reduced vasodilatory effect of insulin suggests vascular insulin resistance. Insulin enhances the expression and activity of eNOS in endothelial cells by upregulating the PI3K/Akt pathway, which induced endothelium-dependent relaxation (Montagnani et al., 2002; Potenza et al., 2009). However, hyperinsulinemia and insulin resistance favor the insulin-dependent activation of MAP-kinases (MAPK), which is associated with the elevated expression of proinflammatory and atherogenic factors (Eringa et al., 2004; Cersosimo et al., 2012; da Silva Franco et al., 2017). The presence of PVAT prevented the reduced insulin-induced relaxation in aorta of LDLr-KO mice. Thus, we hypothesized that an enhanced insulin signaling in PVAT could be a mechanism opposing the vascular insulin resistance in these animals. However, no differences were found for IRS-1, PI3K, and phosphorylated and total Akt and ERK, suggesting that this signaling pathway may not be involved in the protective effect of PVAT. However, we cannot exclude changes in the insulin molecular pathway, other than those evaluated in the present study.

Adiponectin has been described as an ADRF with antiatherogenic properties (Fésüs et al., 2007; Li et al., 2015). The upregulation of adiponectin is observed in PVAT of internal mammary arteries of obese patients with coronary artery disease and might reflect a compensatory mechanism to preserve endothelial function (Cybularz et al., 2017), as previously demonstrated in obese diabetic mice (Liu et al., 2014). In addition, adiponectin improves insulin sensitivity in major insulin target tissues (Ruan and Dong, 2016) and enhances NO production through eNOS activation via PI3K/Akt phosphorylation and eNOS coupling by increasing BH_4_ bioavailability (Margaritis et al., 2013). Thus, we investigated the expression of adiponectin in PVAT and plasma. However, no differences were observed between WT and LDLr-KO mice, suggesting that the effect of PVAT on improving endothelial function in LDLr-KO mice is not associated with modifications of local or circulating levels of this ADRF.

Thoracic PVAT acts as a buffer against toxic levels of fatty acids in arterial circulation and clears fatty acids via inducing thermogenesis (van Dam et al., 2017). Interestingly, enhanced thermogenic activity of PVAT improves endothelial function by inducing the increased release of prostacyclin, whereas impaired PVAT thermogenesis causes atherosclerosis (Chang et al., 2012). Therefore, PVAT adaptive thermogenesis has a beneficial impact on endothelial function protecting against vascular injury. We cannot exclude that this protection exerted by PVAT on endothelial function could be lost in aging. One limitation of the present study is that we did not evaluate PVAT thermogenesis, which is a potential mechanism of endothelial protection in standard-diet fed LDLr-KO mice, as Western-type diets induced whitening and impaired thermogenesis in PVAT (Chang et al., 2013). Recently, Srikakulapu et al. (2017) demonstrated that harboring of B-1 cells by PVAT provides atheroprotection to the aorta, suggesting an additional mechanism for the beneficial role of PVAT in the vasculature.

## Conclusion

Taken together, the results of the present study suggest that aortic PVAT is protective for endothelial dysfunction in LDLr-KO mice, a genetic model of FH. Therefore, this adaptive mechanism in PVAT may protect endothelial function and maintain normal endothelium-dependent relaxation in the early stages of atherosclerotic disease.

## Ethics statement

All experimental protocols were approved (protocol # 3639-1) by the Ethics Committee on Animal Use of the University of Campinas (CEUA-UNICAMP, Campinas-SP, Brazil) and carried out in accordance with the ethical principles for animal experimentation adopted by the Brazilian Society of Laboratory Animal Science (SBCAL/COBEA).

## Author contributions

NB designed and executed the experiments, analyzed the data, wrote the manuscript. DG assisted in the experiments, data analysis and interpretation, read and revised the manuscript. JV performed DAF-2 DA fluorescence analysis. AD conceived the study, guided the experimental design, data analysis and interpretation, read and revised the manuscript.

### Conflict of interest statement

The authors declare that the research was conducted in the absence of any commercial or financial relationships that could be construed as a potential conflict of interest.

## References

[B1] Aghamohammadzadeh R. Unwin R. D. Greenstein A. S. Heagerty A. M. (2016). Effects of obesity on perivascular adipose tissue vasorelaxant function: nitric oxide, inflammation and elevated systemic blood pressure. J. Vasc. Res. 52, 299–305. 10.1159/000443885 26910225 PMC4961268

[B2] Akoumianakis I. Tarun A. Antoniades C. (2017). Perivascular adipose tissue as a regulator of vascular disease pathogenesis: identifying novel therapeutic targets. Br. J. Pharmacol. 174, 3411–3424. 10.1111/bph.13666 27976387 PMC5610156

[B3] Bonfleur M. L. Ribeiro R. A. Balbo S. L. Vanzela E. C. Carneiro E. M. de Oliveira H. C.. (2011). Lower expression of PKAα impairs insulin secretion in islets isolated from low-density lipoprotein receptor (LDLR^−/−^) knockout mice. Metabolism 60, 1158–1164. 10.1016/j.metabol.2010.12.010 21306750

[B4] Brown N. K. Zhou Z. Zhang J. Zeng R. Wu J. Eitzman D. T.. (2014). Perivascular adipose tissue in vascular function and disease: a review of current research and animal models. Arterioscler. Thromb. Vasc. Biol. 34, 1621–1630. 10.1161/ATVBAHA.114.303029 24833795 PMC4104287

[B5] Capettini L. S. Cortes S. F. Silva J. F. Alvarez-Leite J. I. Lemos V. S. (2011). Decreased production of neuronal NOS-derived hydrogen peroxide contributes to endothelial dysfunction in atherosclerosis. Br. J. Pharmacol. 164, 1738–1748. 10.1111/j.1476-5381.2011.01500.x 21615722 PMC3230819

[B6] Cersosimo E. Xu X. Musi N. (2012). Potential role of insulin signaling on vascular smooth muscle cell migration, proliferation, and inflammation pathways. Am. J. Physiol. Cell Physiol. 302, C652–C657. 10.1152/ajpcell.00022.2011 22094332

[B7] Chang L. Milton H. Eitzman D. T. Chen Y. E. (2013). Paradoxical roles of perivascular adipose tissue in atherosclerosis and hypertension. Circ. J. 77, 11–18. 10.1253/circj.CJ-12-1393 23207957

[B8] Chang L. Villacorta L. Li R. Hamblin M. Xu W. Dou C.. (2012). Loss of perivascular adipose tissue on peroxisome proliferator-activated receptor-γ deletion in smooth muscle cells impairs intravascular thermoregulation and enhances atherosclerosis. Circulation 126, 1067–1078. 10.1161/CIRCULATIONAHA.112.104489 22855570 PMC3493564

[B9] Chatterjee T. K. Stoll L. L. Denning G. M. Harrelson A. Blomkalns A. L. Idelman G.. (2009). Proinflammatory phenotype of perivascular adipocytes: influence of high-fat feeding. Circ. Res. 104, 541–549. 10.1161/CIRCRESAHA.108.182998 19122178 PMC2742882

[B10] Creager M. A. Cooke J. P. Mendelsohn M. E. Gallagher S. J. Coleman S. M. Loscalzo J.. (1990). Impaired vasodilation of forearm resistance vessels in hypercholesterolemic humans. J. Clin. Invest. 86, 228–234. 10.1172/JCI114688 2195060 PMC296711

[B11] Creager M. A. Gallagher S. J. Girerd X. J. Coleman S. M. Dzau V. J. Cooke J. P. (1992). L-arginine improves endothelium-dependent vasodilation in hypercholesterolemic humans. J. Clin. Invest. 90, 1248–1253. 10.1172/JCI115987 1401062 PMC443166

[B12] Cybularz M. Langbein H. Zatschler B. Brunssen C. Deussen A. Matschke K.. (2017). Endothelial function and gene expression in perivascular adipose tissue from internal mammary arteries of obese patients with coronary artery disease. Atheroscler. Suppl. 30, 149–158. 10.1016/j.atherosclerosissup.2017.05.042 29096831

[B13] da Silva Franco N. Lubaczeuski C. Guizoni D. M. Victorio J. A. Santos-Silva J. C. Brum P. C.. (2017). Propranolol treatment lowers blood pressure, reduces vascular inflammatory markers and improves endothelial function in obese mice. Pharmacol. Res. 122, 35–45. 10.1016/j.phrs.2017.05.018 28539257

[B14] Davel A. P. Ceravolo G. S. Wenceslau C. F. Carvalho M. H. Brum P. C. Rossoni L. V. (2012). Increased vascular contractility and oxidative stress in β2-Adrenoceptor knockout mice: the role of NADPH oxidase. J. Vasc. Res. 49, 342–352. 10.1159/000337486 22627472

[B15] Dorighello G. G. Paim B. A. Kiihl S. F. Ferreira M. S. Catharino R. R. Vercesi A. E.. (2016). Correlation between mitochondrial reactive oxygen and severity of atherosclerosis. Oxidative Med. Cell. Longev. 2016:7843685. 10.1155/2016/7843685 26635912 PMC4655284

[B16] Dorighello G. G. Paim B. A. Leite A. C. Vercesi A. E. Oliveira H. C. (2017). Spontaneous experimental atherosclerosis in hypercholesterolemic mice advances with ageing and correlates with mitochondrial reactive oxygen species. Exp. Gerontol. [Epub ahead of print]. 10.1016/j.exger.2017.02.010 28213051

[B17] Eringa E. C. Stehouwer C. D. van Nieuw Amerongen G. P. Ouwehand L. Westerhof N. Sipkema P. (2004). Vasoconstrictor effects of insulin in skeletal muscle arterioles are mediated by ERK1/2 activation in endothelium. Am. J. Physiol. Heart Circ. Physiol. 287, H2043–H2048. 10.1152/ajpheart.00067.2004 15059773

[B18] Feron O. Dessy C. Moniotte S. Desager J. P. Balligand J. L. (1999). Hypercholesterolemia decreases nitric oxide production by promoting the interaction of caveolin and endothelial nitric oxide synthase. J. Clin. Invest. 103, 897–905. 10.1172/JCI4829 10079111 PMC408139

[B19] Fésüs G. Dubroska G. Gorzelnial K. Kluge R. Huang Y. Luft F. C.. (2007). Adiponectin is a novel humoral vasodilator. Cardiovasc. Res. 75, 719–727. 10.1016/j.cardiores.2007.05.025 17617391

[B20] Gil-Ortega M. Stucchi P. Guzmán-Ruiz R. Cano V. Arribas S. González M. C.. (2010). Adaptative nitric oxide overproduction in perivascular adipose tissue during early diet-induced obesity. Endocrinology 151, 3299–3306. 10.1210/en.2009-1464 20410199

[B21] Guizoni D. M. Dorighello G. G. Oliveira H. C. Delbin M. A. Krieger M. H. Davel A. P. (2016). Aerobic exercise training protects against endothelial dysfunction by increasing nitric oxide and hydrogen peroxide production in LDL receptor-deficient mice. J. Transl. Med. 14:213. 10.1186/s12967-016-0972-z 27435231 PMC4950099

[B22] Guzik T. J. West N. E. Black E. McDonald D. Ratnatunga C. Pillai R.. (2000). Vascular superoxide production by NAD(P)H oxidase: association with endothelial dysfunction and clinical risk factors. Circ. Res. 86, e85–e90. 10.1161/01.RES.86.9.e85 10807876

[B23] Heitzer T. Schlinzig T. Krohn K. Meinertz T. Münzel T. (2001). Endothelial dysfunction, oxidative stress, and risk of cardiovascular events in patients with coronary artery disease. Circulation 104, 2673–2678. 10.1161/hc4601.099485 11723017

[B24] Hofmann A. Brunssen C. Poitz D. M. Langbein H. Strasser R. H. Henle T.. (2017). Lectin-like oxidized low-density lipoprotein receptor-1 promotes endothelial dysfunction in LDL receptor knockout background. Atheroscler. Suppl. 30, 294–302. 10.1016/j.atherosclerosissup.2017.05.020 29096854

[B25] Kauser K. da Cunha V. Fitch R. Mallari C. Rubanyi G. M. (2000). Role of endogenous nitric oxide in progression of atherosclerosis in apolipoprotein E-deficient mice. Am. J. Physiol. Heart Circ. Physiol. 278, H1679–H1685. 10.1152/ajpheart.2000.278.5.H1679 10775149

[B26] Ketonen J. Shi J. Martonen E. Mervaala E. (2010). Periadventitial adipose tissue promotes endothelial dysfunction via oxidative stress in diet-induced obese C57Bl/6 mice. Circ. J. 74, 1479–1487. 10.1253/circj.CJ-09-0661 20526041

[B27] Langbein H. Brunssen C. Hofmann A. Cimalla P. Brux M. Bornstein S. R.. (2016). NADPH oxidase 4 protects against development of endothelial dysfunction and atherosclerosis in LDL receptor deficient mice. Eur. Heart J. 37, 1753–1761. 10.1093/eurheartj/ehv564 26578199 PMC4900759

[B28] Langbein H. Hofmann A. Brunssen C. Goettsch W. Morawietz H. (2015). Impact of high-fat diet and voluntary running on body weight and endothelial function in LDL receptor knockout mice. Atheroscler. Suppl. 18, 59–66. 10.1016/j.atherosclerosissup.2015.02.010 25936306

[B29] Li C. Wang Z. Wang C. Ma Q. Zhao Y. (2015). Perivascular adipose tissue-derived adiponectin inhibits collar-induced carotid atherosclerosis by promoting macrophage autophagy. PLoS ONE 10:e0124031. 10.1371/journal.pone.0124031 26020520 PMC4447395

[B30] Li Y. Mihara K. Saifeddine M. Krawetz A. Lau D. C. Li H. (2011). Perivascular adipose tissue-derived relaxation factors: release by peptide agonists via proteinase-activated receptor-2 (PAR2) and non-PAR2 mechanisms. Br. J. Pharmacol. 164, 1990–2002. 10.1111/j.1476-5381.2011.01501.x 21615723 PMC3246662

[B31] Liu Y. Li D. Zhang Y. Sun R. Xia M. (2014). Anthocyanin increases adiponectin secretion and protects against diabetes-related endothelial dysfunction. Am. J. Physiol. Endocrinol. Metab. 306, E975–E988. 10.1152/ajpendo.00699.2013 24595303

[B32] Margaritis M. Antonopoulos A. S. Digby J. Lee R. Reilly S. Coutinho P.. (2013). Interactions between vascular wall and perivascular adipose tissue reveal novel roles for adiponectin in the regulation of endothelial nitric oxide synthase function in human vessels. Circulation 127, 2209–2221. 10.1161/CIRCULATIONAHA.112.001133 23625959

[B33] Moe K. T. Naylynn T. M. Yin N. O. Khairunnisa K. Allen J. C. Wong M. C.. (2013). Tumor necrosis factor-alpha induces aortic intima-media thickening via perivascular adipose tissue inflammation. J. Vasc. Res. 50, 228–237. 10.1159/000350542 23711955

[B34] Montagnani M. Ravichandran L. V. Chen H. Esposito D. L. Quon M. J. (2002). Insulin receptor substrate-1 and phosphoinositide-dependent kinase-1 are required for insulin-stimulated production of nitric oxide in endothelial cells. Mol. Endocrinol. 16, 1931–1942. 10.1210/me.2002-0074 12145346

[B35] Mozaffarian D. Benjamin E. J. Go A. S. Arnett D. K. Blaha M. J. Cushman M.. (2016). Heart disease and stroke statistics-2016 update: a report from the American Heart Association. Circulation 133, e38–360. 10.1161/CIR.0000000000000366 26673558

[B36] Öhman M. K. Luo W. Wang H. Guo C. Abdallah W. Russo H. M.. (2011). Perivascular visceral adipose tissue induces atherosclerosis in apolipoprotein E deficient mice. Atherosclerosis 219, 33–39. 10.1016/j.atherosclerosis.2011.07.012 21835408 PMC3206153

[B37] Potenza M. A. Addabbo F. Montagnani M. (2009). Vascular actions of insulin with implications for endothelial dysfunction. Am. J. Physiol. Endocrinol. Metab. 297, E568–E577. 10.1152/ajpendo.00297.2009 19491294

[B38] Rabelo L. A. Cortes S. F. Alvarez-Leite J. I. Lemos V. S. (2003). Endothelium dysfunction in LDL receptor knockout mice: a role for H_2_O_2_. Br. J. Pharmacol. 138, 1215–1220. 10.1038/sj.bjp.0705164 12711621 PMC1573774

[B39] Ruan H. Dong L. Q. (2016). Adiponectin signaling and function in insulin target tissues. J. Mol. Cell Biol. 8, 101–109. 10.1093/jmcb/mjw014 26993044 PMC4816150

[B40] Siasos G. Tousoulis D. Antoniades C. Stefanadi E. Stefanadis C. (2007). L-Arginine, the substrate for NO synthesis: an alternative treatment for premature atherosclerosis? Int. J. Cardiol. 116, 300–308. 10.1016/j.ijcard.2006.04.062 16860889

[B41] Souza J. C. Vanzela E. C. Ribeiro R. A. Rezende L. F. de Oliveira C. A. Carneiro E. M.. (2013). Cholesterol reduction ameliorates glucose-induced calcium handling and insulin secretion in islets from low-density lipoprotein receptor knockout mice. Biochim. Biophys. Acta 1831, 769–775. 10.1016/j.bbalip.2012.12.013 23298460

[B42] Srikakulapu P. Upadhye A. Rosenfeld S. M. Marshall M. A. McSkimming C. Hickman A. W. (2017). Perivascular adipose tissue harbors atheroprotective IgM-producing B cells. Front Physiol. 8:719. 10.3389/fphys.2017.00719 28970806 PMC5609437

[B43] Stapleton P. A. Goodwill A. G. James M. E. Brock R. W. Frisbee J. C. (2010). Hypercholesterolemia and microvascular dysfunction: interventional strategies. J. Inflamm. 7:54. 10.1186/1476-9255-7-54 21087503 PMC2996379

[B44] Takaoka M. Suzuki H. Shioda S. Sekikawa K. Saito Y. Nagai R.. (2010). Endovascular injury induces rapid phenotypic changes in perivascular adipose tissue. Arterioscler. Thromb. Vasc. Biol. 30, 1576–1582. 10.1161/ATVBAHA.110.207175 20489168

[B45] van Dam A. D. Boon M. R. Berbée J. F. P. Rensen P. C. N. van Harmelen V. (2017). Targeting white, brown and perivascular adipose tissue in atherosclerosis development. Eur. J. Pharmacol. 816, 82–92. 10.1016/j.ejphar.2017.03.051 28347739

[B46] Vanhoutte P. M. (2003). Endothelial control of vasomotor function: from health to coronary disease. Circ. J. 67, 572–575. 10.1253/circj.67.572 12845177

[B47] Victorio J. A. Fontes M. T. Rossoni L. V. Davel A. P. (2016). Different anti-contractile function and nitric oxide production of thoracic and abdominal perivascular adipose tissues. Front. Physiol. 7:295. 10.3389/fphys.2016.00295 27462277 PMC4940415

[B48] World Health Organization (2014). The Top 10 Causes of Death. Fact Sheet N°310. Available online at: http://www.whoint/mediacentre/factsheets/fs310/en/index.html

[B49] Wu H. Y. Jeng Y. Y. Yue C. J. Chyu K. Y. Hsueh W. A. Chan T. M. (1994). Endothelial-dependent vascular effects of insulin and insulin-like growth factor I in the perfused rat mesenteric artery and aortic ring. Diabetes 43, 1027–1032. 10.2337/diab.43.8.1027 8039596

[B50] Xia N. Horke S. Habermeier A. Closs E. I. Reifenberg G. Gericke A.. (2016). Uncoupling of endothelial nitric oxide synthase in perivascular adipose tissue of diet-induced obese mice. Arterioscler. Thromb. Vasc. Biol. 36, 78–85. 10.1161/ATVBAHA.115.306263 26586660

[B51] Zhou X. He P. (2011). Improved measurements of intracellular nitric oxide in intact microvessels using 4,5-diaminofluorescein diacetate. Am. J. Physiol. Heart Circ. Physiol. 301, H108–H114. 10.1152/ajpheart.00195.2011 21536843 PMC3129913

